# A transcriptomic analysis of serial-cultured, tonsil-derived mesenchymal stem cells reveals decreased integrin α3 protein as a potential biomarker of senescent cells

**DOI:** 10.1186/s13287-020-01860-y

**Published:** 2020-08-17

**Authors:** Da Hyeon Choi, Se-Young Oh, Ju Kwang Choi, Kyeong Eun Lee, Ju Yeon Lee, Yoon Jeong Park, Inho Jo, Yoon Shin Park

**Affiliations:** 1 Department of Microbiology, School of Biological Sciences, College of Natural Sciences, Chungbuk National University, Cheongju, 28644 Republic of Korea; 2 Department of Molecular Medicine, College of Medicine, Ewha Womans University, Seoul, 07804 Republic of Korea; 3 Ewha Tonsil-derived Mesenchymal Stem Cells Research Center (ETSRC), College of Medicine, Ewha Womans University, Seoul, 07804 Republic of Korea; 4 Central Research Institute, Nano Intelligent Biomedical Engineering Corporation (NIBEC), School of Dentistry, Seoul National University, Seoul, 03080 Republic of Korea; 5 Department of Dental Regenerative Bioengineering and Dental Research Institute, School of Dentistry, Seoul National University, Seoul, 03080 Republic of Korea

**Keywords:** AKT, Culture-aged, ECM-receptor protein, Integrin α3, Senescence, Serial passaging, Tonsil-derived mesenchymal stem cells, Transcriptome

## Abstract

**Background:**

Mesenchymal stem cells (MSCs) have been widely used for stem cell therapy, and serial passage of stem cells is often required to obtain sufficient cell numbers for practical applications in regenerative medicine. A long-term serial cell expansion can potentially induce replicative senescence, which leads to a progressive decline in stem cell function and stemness, losing multipotent characteristics. To improve the therapeutic efficiency of stem cell therapy, it would be important to identify specific biomarkers for senescent cells.

**Methods:**

Tonsil-derived mesenchymal stem cells (TMSCs) with 20–25 passages were designated as culture-aged TMSCs, and their mesodermal differentiation potentials as well as markers of senescence and stemness were compared with the control TMSCs passaged up to 8 times at the most (designated as young). A whole-genome analysis was used to identify novel regulatory factors that distinguish between the culture-aged and control TMSCs. The identified markers of replicative senescence were validated using Western blot analyses.

**Results:**

The culture-aged TMSCs showed longer doubling time compared to control TMSCs and had higher expression of senescence-associated (SA)-β-gal staining but lower expression of the stemness protein markers, including Nanog, Oct4, and Sox2 with decreased adipogenic, osteogenic, and chondrogenic differentiation potentials. Microarray analyses identified a total of 18,614 differentially expressed genes between the culture-aged and control TMSCs. The differentially expressed genes were classified into the Gene Ontology categories of cellular component (CC), functional component (FC), and biological process (BP) using KEGG (Kyoto encyclopedia of genes and genomes) pathway analysis. This analysis revealed that those genes associated with CC and BP showed the most significant difference between the culture-aged and control TMSCs. The genes related to extracellular matrix-receptor interactions were also shown to be significantly different (*p* < 0.001). We also found that culture-aged TMSCs had decreased expressions of integrin α3 (ITGA3) and phosphorylated AKT protein (p-AKT-Ser^473^) compared to the control TMSCs.

**Conclusions:**

Our data suggest that activation of ECM-receptor signaling, specifically involved with integrin family-mediated activation of the intracellular cell survival-signaling molecule AKT, can regulate stem cell senescence in TMSCs. Among these identified factors, ITGA3 was found to be a representative biomarker of the senescent TMSCs. Exclusion of the TMSCs with the senescent TMSC markers in this study could potentially increase the therapeutic efficacy of TMSCs in clinical applications.

## Background

The differentiation efficacy of mesenchymal stem cells (MSCs) is relatively restricted compared with that of the pluripotent stem cells, such as embryonic stem cells (ESCs) and induced pluripotent stem cells (iPSCs) [1, 2]. Nevertheless, MSCs are generally considered as a more practical option for cell therapies since transplanted stem cells derived from MSCs have a much lower risk of forming tumors and are relatively safer compared with pluripotent stem cells [1, 3]. Regardless of their therapeutic potential and safety, MSCs must be serially passaged for an extended period to obtain a sufficient cell number for in vivo transplantation or clinical application [4–7]. With continuous serial passages, MSCs gradually lose their self-renewal, stemness, and regenerative potential [8]. Senescent MSCs secrete numerous factors that decrease proliferation [8] and migration of a stem cell [9–11] but increase inflammatory responses [12]. Notably, during long-term cell culture, MSCs may undergo molecular changes that result in the acquisition of senescent phenotypes [9, 10] independent of MSC isolation and culture conditions [13].

Human tonsil-derived MSCs (TMSCs) are obtained from discarded children’s tonsillar tissues after tonsillectomy. TMSCs have been considered as a potential therapeutic tool in the application of tissue engineering and regenerative medicine [14–16], because of their high regenerative capacity and multipotency to differentiate into various cell types, such as the bone [17], cartilage [17], adipose [18], muscle [19, 20], tendon [21], stroma, and neuronal cells [22, 23]. Unfortunately, a long-term culture of TMSCs could lead to replicative senescence, decreasing the stemness and multi-differentiation potential of TMSCs. Senescence is a phenomenon that has been implicated in the loss of stemness, which ultimately leads to a gradual decrease in proliferation potential and impaired function [4, 24]. Understanding the molecular processes that regulate TMSC proliferation and commitment to specific cell lineages is crucial in determining potential dysfunction of TMSCs with replicative senescence for effective application of MSCs in a clinical setting [13, 25].

MSCs undergo continuous self-renewal throughout an organism’s lifespan. The complex milieu composed of cells and extracellular matrix (ECM), as well as signaling molecules associated with stem cells, are collectively referred to as a stem cell niche [10, 26]. Stem cell self-renewal is tightly regulated by the concerted action of intrinsic factors and signals from stem cells within the niche. The signals from the niche often function within a short-range, allowing the cells within the niche to self-renew, while their daughters outside the niche differentiate to specific cell types [27, 28]. Thus, for stem cells to continuously self-renew, they are often anchored in the niche via adhesion molecules [29]. Age-related changes in the composition and structure of ECM anchoring proteins have a strong impact on the regenerative process [30, 31].

Aging of stem cells could likely decrease the expression of cell adhesion molecules, such as members of the integrin family and related signaling pathways [29]. Integrins are heterodimeric cell adhesion receptors formed by an “*α*” and a “*β*” subunit [32]. They recognize changes in the extracellular environment and regulate intracellular signaling and membrane-bounded organelles [33]. The integrins mediate major downstream signaling pathways involved in regulating cellular phenotypes, such as migration, adhesion, and proliferation. Because the integrins are such important regulators of cellular processes, they play pivotal roles under different pathophysiological conditions, such as development and aging [34].

In this study, we investigated differential gene expression patterns among TMSCs with different cultivation times and passage numbers. Transcriptomic approaches were used to further identify novel candidate biomarkers of culture-aged, senescent TMSCs. These analyses have led us to propose that genes associated with ECM are differentially altered with the aging of TMSCs, and some of these molecules could be used as potential indicators for identifying stem cells with replicative senescence [10, 35].

## Methods

### Isolation and culture of TMSCs

TMSCs were isolated from tonsillar tissue removed during a tonsillectomy, as it was previously described [17]. Removed tonsil tissues were collected and further used for TMSC isolation according to the guidelines of the Ewha Womans University Medical Center (EWUMC, IRB No. ECT-11-53-02). Informed written consent was obtained from legal guardians of all patients participating in this study, and the study protocol was approved by the EWUMC institutional review board. Briefly, tonsillar tissues were acquired from four donors (2 girls and 2 boys) who had undergone a tonsillectomy. Tonsillar tissues used in this experiment were collected from the patients who had taken tonsillectomy due to tonsil hyperplasia without tonsillitis as well as other diseases. Donors who enrolled in this experiment were under 10 years of age. The isolated tonsils were mechanically digested by cutting, mincing, and grinding, and the tissues were then enzymatically digested with collagenase type I (Thermo Fisher Scientific, Waltham, MA, USA) and DNase (Sigma-Aldrich, St. Louis, MO, USA) at 37 °C for 30 min. The obtained cell suspension was filtered through a wire mesh, and mononuclear cells were isolated using Ficoll-Paque (GE Healthcare, Piscataway, NJ, USA) density gradient centrifugation. The resulting mononuclear cells were cultured in DMEM-HG (Welgene Inc., Gyeongsan, Korea) supplemented with 10% fetal bovine serum (FBS: certified, US origin, Gibco, Grand Island, NY, USA), 1% antibiotics/antimycotics (A/A), and penicillin/streptomycin (P/S) (Gibco) at 37 °C in a humidified 5% CO_2_ incubator. Cells were allowed to adhere to culture plates for 24 h, and adherent mononuclear cells were taken as TMSCs. The cells were cultured in DMEM supplemented with 10% FBS, and the media was changed every 2 days. When the TMSCs reached 80–90% confluent, they were treated with 0.25% trypsin-EDTA (Gibco) for 3 min. The detached cells were washed with PBS twice and collected by centrifuging at 3000 rpm for 5 min (Eppendorf, Hamburg, Germany).

### Experimental groups of TMSCs

After reaching 80% confluence, TMSCs were subcultured onto individual dishes and serially cultured to passages 20 to 25 to induce senescence. The maximum passage number was determined by the proliferative capacity. TMSCs used for the current study were between passages 5 and 25. The growth medium was changed every 3 days. In vitro-cultured TMSCs were divided into two groups as follows: control TMSCs (passages 5–8) and culture-aged TMSCs (passages 20–25). Morphological changes in the TMSCs were monitored daily by examination under an inverted microscope (Olympus, Tokyo, Japan). Doubling time of TMSCs with different passage numbers was determined using the following Patterson formula: doubling time (h) = [{(T − T0)(log_2_)}/(logN − logN0)], where *T* is time (h) and *N* is the cell count.

### Fluorescence-activated cell sorting (FACS) analysis

TMSCs were phenotypically characterized by flow cytometry. The TMSCs (1.0 × 10^4^ cells) from the two experimental groups were incubated with fluorescein isothiocyanate (FITC)- or phycoerythrin (PE)-conjugated monoclonal antibodies against Isotype-PE, Isotype-FITC, CD14, CD34, CD45, CD73, CD90, and CD105 (BD Biosciences, San Jose, CA, USA) for 30 min at 4 °C. The cell populations were analyzed using a FACScan instrument (FACSCalibur-S System; BD Biosciences). A total of about 1 × 10^4^ cells were counted, of which 9832 were live cells except of dead cell and debris. As a control, non-treatment TMSCs and isotype-PE and isotype-FITC Ig control for each wavelength were used. Data were analyzed using Flowjo (BD Biosciences). Results were displayed as the percentage of cells labeled for each monoclonal antibody.

### Senescence-associated-β-gal assay

Morphological changes associated with experimental treatments, including increased cell size, altered overall morphology, and decreased proliferative capacity, were assessed with an inverted microscope (Olympus). Senescent TMSCs were detected by senescence-associated β-galactosidase (SA-β-gal) staining using an SA-β-gal staining kit (Cell Signaling Technology, Boston, MA, USA) according to the manufacturer’s instructions. Briefly, TMSCs were fixed with 4% paraformaldehyde (PFA) (Biosesang, Seongnam, Korea) for 15 min at room temperature and then were incubated overnight with β-gal staining solution at 37 °C in a dry incubator without a CO_2_ supply. Culture-aged cells were identified by their blue staining of β-gal solution under a standard light microscope. The culture-aged cells were expressed as a percentage of total TMSCs.

### Changes in multipotential differentiation of TMSCs

Changes in mesodermal differentiation potentials of TMSCs with senescence were assessed by incubating TMSCs with adipogenic, osteogenic, or chondrogenic differentiation medium (Thermo Fisher Scientific) for 3 weeks. Thereafter, adipogenic-, osteogenic-, and chondrogenic-differentiated TMSCs were washed twice with Dulbecco’s phosphate-buffered saline (DPBS) and then fixed with 4% PFA for 15 min at room temperature. The fixed, differentiated cells were washed with PBS, then stained with 2% Oil Red O, 2% Alizarin Red S, or 1% Alcian Blue solution (Sciencell, Carlsbad, USA) for 1 h at room temperature to determine levels of adipogenicity, osteogenicity, or chondrogenicity, respectively. Adipogenic differentiation capacity was quantified by assessing lipid accumulation by eluting Oil Red O deposited in adipogenic-differentiated TMSCs with 100% isopropanol for 10 min and measuring the absorbance of the eluted solution at a wavelength of 540 nm using a microplate reader (Synergy HTX, BioTec, Seoul, Korea). Calcium deposition in osteogenic-differentiated TMSCs was quantified by eluting Alizarin Red S stain by incubating stained cells with 10% cetylpyridinium chloride (Sigma-Aldrich) for 10 min. The eluate was collected and its absorbance at a wavelength of 570 nm was measured. Chondrogenic differentiation was quantified by solubilizing Alcian Blue-stained cells with 6 M guanidine hydrochloride (Sigma-Aldrich) and measuring the absorbance of the elute at a wavelength of 605 nm.

### RNA quality assessment

TMSCs from a total of four donors (2 boys and 2 girls) with different passage numbers were used. RNA used for microarray analysis was obtained from control (5 passages) and culture-aged (25 passages) from each of two donors (*n* = 2 per each passage group). The RNA was isolated from the TMSCs using TRIzol reagent (Thermo Fisher Scientific). RNA purity (260/280 ratio) and RNA integrity number (RIN) were evaluated using an ND-1000 spectrophotometer (NanoDrop, Wilmington, USA) and Agilent 2100 Bioanalyzer (Agilent Technologies, Palo Alto, USA), respectively. The RNA samples with higher than 1.7 for RNA purity and 7.0 for RIN were used for the microarray analyses.

### Affymetrix whole transcriptomic arrays

The Affymetrix whole transcript expression array process was executed according to the manufacturer’s protocol (GeneChip Whole Transcript PLUS Reagent Kit). cDNA was synthesized using the GeneChip WT Amplification kit, as described by the manufacturer. The sense cDNA was then fragmented and biotin-labeled with TdT (terminal deoxynucleotidyl transferase) using the GeneChip WT terminal labeling kit. Approximately 5.5 μg of labeled cDNA was hybridized to the Affymetrix GeneChip, Human Clariom D array, at 45 °C for 16 h. Hybridized arrays were washed and stained on a GeneChip fluidics station 450 and scanned on a GCS3000 scanner (Thermo Fisher Scientific). Signal values were computed using Affymetrix GeneChip Command console software 5.0 (Thermo Fisher Scientific). Data can be found via GEO accession number GES149588.

### Preparation and statistical analysis of transcriptomic data

Data were summarized and normalized using the robust multi-average (RMA) method, implemented in Affymetrix Power Tools. Results of gene-level RMA analyses were exported for analysis of differentially expressed genes (DEGs). The statistical significance of expression data was determined using a local-pooled-error test and measurements of fold change (fc), with the null hypothesis being that no difference exists between groups. False discovery rate (FDR) was controlled by adjusting the *P* value using the Benjamini-Hochberg algorithm. For a given DEG set, hierarchical cluster analysis was performed using complete linkage and Euclidean distance as a measure of similarity.

Gene-Enrichment and Functional Annotation analyses of significant probe lists were performed using Gene Ontology (http://geneontology.org) and KEGG (http://kegg.jp). All data analyses and visualization of differentially expressed genes were conducted using R 3.0.2 (www.r-project.org).

### Western blot analysis

For Western blotting, TMSCs were lysed with a lysis buffer (20 mM Tris-HCl pH 7.5, 150 mM NaCl, 1% Triton X-100, 1 mM EDTA, 1 mM EGTA, 1 mM phenylmethylsulfonyl fluoride, 10 mM β-glycerophosphate, 1 mM NaF, and 1 mM Na_3_VO_4_) containing Protease Inhibitor Mixture (Roche Applied Science, Mannheim, Germany). Equal amounts of protein (30 μg) were separated by sodium dodecyl sulfate polyacrylamide gel electrophoresis (SDS-PAGE) on 12% gels and transferred onto nitrocellulose membranes (GE Healthcare). After blocking the membranes in TBST with 5% skim milk, the blots were incubated with appropriate primary antibodies followed by the corresponding secondary antibodies, then developed using enhanced chemiluminescence (ECL) reagents. Primary antibodies against the following proteins were used in this study: Nanog, Sox2, Oct4, integrin α3, integrin α8, and integrin β1 (Abcam, Cambridge, MA, UK); AKT, p-AKT-Ser^473^, and p-AKT-Thr^308^ (Cell Signaling Technology, Beverly, MA, USA); and GAPDH (AB Frontier Inc., Seoul, Korea). All protein band images from Western blot analyses were quantified densitometrically using ImageJ software (National Institutes of Health, Bethesda, MD, USA).

### Statistical analysis

All data are presented as means ± standard deviation (S.D.). The statistical significance of differences among varying TMSC passage numbers (5, 10, 15, 20, and 25) were determined using one-way analysis of variance (ANOVA). The significance of differences between two experimental groups (control and culture-aged TMSCs) was analyzed using Student’s *t* test. A *P* value < 0.05 was considered statistically significant.

## Results

### Confirmation of culture-aged senescent TMSCs

**Fig. 1:**
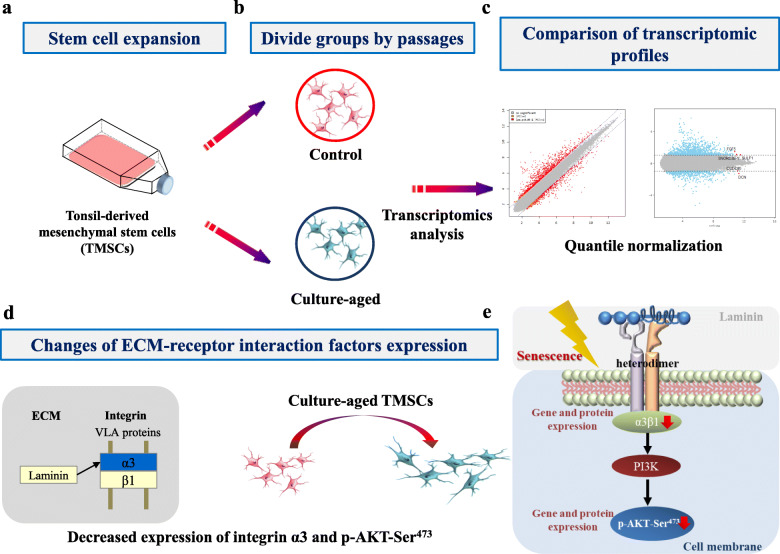
Schematic illustration of the current study. A schematic diagram illustrating. **a** TMSCs were isolated and serially cultured until passage 20 to 25 to obtain culture-aged senescent TMSC. **b** TMSCs were divided into two experimental groups according to passage number: control TMSCs (passage number 5–8) and culture-aged TMSCs (passage number 20–25). **c** Transcriptomic microarray analysis of differential gene expression between control and culture-aged TMSCS .**d** ECM-receptor interaction-related genes were identified by KEGG pathway analysis. Of these genes, *ITGA3* was selected as a representative biomarker for the TMSCs with replicative senescence. **e** Graphical summary of the ITGA3-AKT signaling pathway

A schematic depiction of the procedure used in this experiment is illustrated in Fig. 1. The isolated TMSCs from patients who had undergone a tonsillectomy were serially cultured (Fig. 1a) and divided into two groups based on the number of passages (Fig. 1b). TMSCs with a passage number between 5 and 8 were categorized as the control group and those that were serially passaged from 20 to 25 times were designated as culture-aged TMSCs (Fig. 1b). The obtained culture-aged TMSCs were quantified by quantile normalization, and differences in genetic/transcriptomic expression profiles between control and culture-aged TMSC groups were examined (Fig. 1c). A functional KEGG (Kyoto Encyclopedia of Genes and Genomes) pathway analysis revealed differentially enriched signaling pathways between the two TMSC groups (Fig. 1d). The schematic illustration of the interactions between integrin and the AKT pathway determined in the present study are shown in Fig. 1e.

**Fig. 2:**
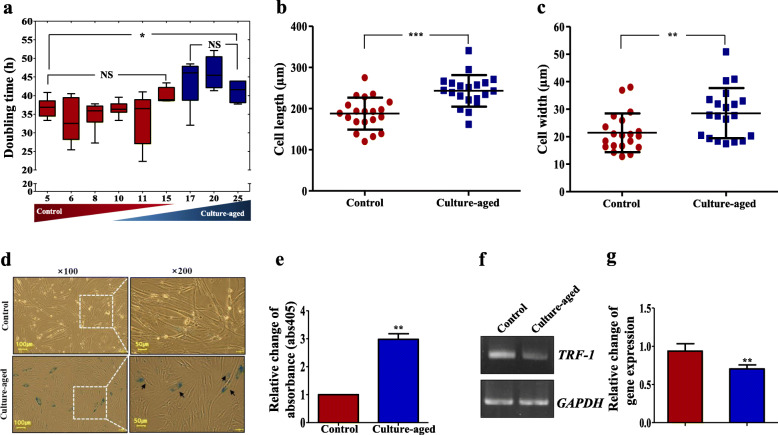
Changes in senescent markers with serial passaging of TMSCs. **a** Doubling times were calculated at each passage of TMSCs using Patterson formula. Morphological changes of control and culture-aged TMSC groups were determined by measuring cell length (**b**) and width (**c**). Control TMSCs exhibited a polygonal morphology, whereas culture-aged TMSCs formed dispersed shapes. **d** Senescent cells were identified as blue-stained cells under an optical microscope. Magnification: **×** 40, **×** 100, and **×** 200; scale bar, 200 μm. White dotted boxes indicate magnified images of the same region. **e** The average absorbance intensity of SA-β-gal staining (405 nm) from 5 randomly selected fields between control and culture-aged TMSCs were compared. **f** Gene expression of TRF-1 in control and culture-aged TMSCs. **g** Bar graph indicates relative fold changes in the expression of TRF-1. The *p* values were considered statistically significant at the *p* < 0.05 (*), *p* < 0.01 (**), and *p* < 0.001 (***), and significant differences among experimental groups were indicated with different alphabetical letters in figures

Changes in the doubling time of TMSCs with different passage numbers were also assessed (Fig. 2a). The doubling time within 10 passages were determined to be 38 h, which was taken as the control in this study. Maximum doubling time for the culture-aged TMSCs was determined to be to 51 h (Fig. 2a).

The culture-aged TMSCs were confirmed by SA-β gal assay as well as by assessing morphological changes (Fig. 2). Culture-aged TMSCs were significantly longer (243.13 ± 38.42 vs. 28.54 ± 9.11 μm) and wider (187.88 ± 38.74 vs. 21.43 ± 7.04 μm) than the young control TMSCs (Fig. 2b, c). Statistical analyses further confirmed these results, showing that the cell size (*p* < 0.001) and diameter (*p* < 0.01) of the culture-aged TMSCs were significantly larger in compared with those of the control group (Fig. 2b, c). SA-β-gal staining revealed a higher intensity of X-gal staining among the culture-aged TMSCs compared with that in the control group (Fig. 2d). A quantitative analysis of the culture-aged TMSCs revealed approximately 3.0 ± 0.2-fold increase in X-gal staining (*p* < 0.01) (Fig. 2e). Reverse transcriptase PCR analysis showed that the culture-aged TMSCs had significantly lower gene expression of TRF-1 compared to the control TMSCs (Fig. 2f, g; *p* < 0.01).

### Decreased stemness and multi-differentiation potential

**Fig. 3:**
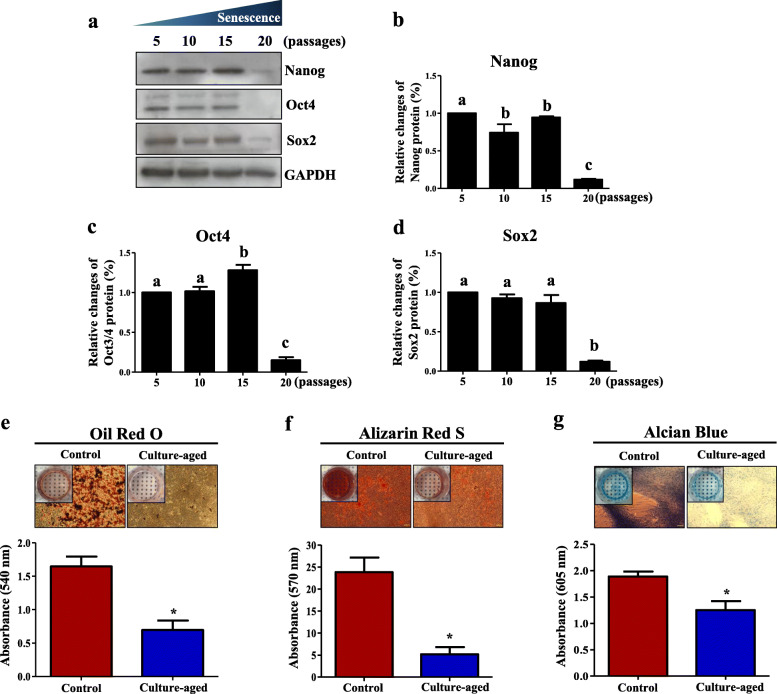
Decreased stemness and multi-differentiation potential of TMSCs upon serial passaging. **a** Protein expression of the embryonic stem cell markers, Nanog, Oct4, and Sox2, in TMSCs was investigated using Western blot analyses. **b**–**d** Bar graph represents the band intensity of each marker normalized to the intensity of the respective GAPDH band. Significant differences among experimental groups are indicated with different alphabetical letters in figures. **e**–**g** Changes in mesodermal differentiation potentials of TMSCs with serial passaging. Multi-differentiation potential between control and culture-aged TMSCs was compared by assessing adipogenesis, osteogenesis, and chondrogenesis. The differentiated TMSCs were stained with Oil Red O (**e**), Alizarin Red S (**f**), or Alcian Blue (**g**). The intracellular staining was extracted, and absorbance of the extracts was measured to compare differentiation potential among different TMSC groups. Bar graph represents the optical density of extracts from cells stained with Oil Red O (adipogenesis), Alizarin Red S (osteogenesis), or Alcian Blue (chondrogenesis), determined the absorbance at wavelengths of 540, 570, and 605 nm, respectively (*p < 0.05)

We next investigated changes in expression of the stem cell markers, Nanog, Oct4, and Sox2, between the control and culture-aged TMSCs. Protein expression of stemness markers was maintained until the 15th passage, but significantly decreased until the 20th passage (Fig. 3a–d).

We also examined the effect of the culture-aged senescence on mesodermal differentiation potentials into adipocyte, osteocyte, and chondrocyte (Fig. 3e–g). Compared with the control TMSCs, the culture-aged TMSCs showed significantly lower mesodermal differentiation potentials. Absorbance values of eluates of Oil Red O-, Alizarin Red S-, and Alcian Blue-stained culture-aged TMSCs decreased from 1.6 ± 0.1 to 0.7 ± 0.1 (Fig. 3e), 25.0 ± 3.7 to 5.0 ± 2.8 (Fig. 3f), and 2.0 ± 0.3 to 1.4 ± 0.5 (Fig. 3 g), respectively, compared with the control TMSCs. The results indicate that replicative senescence induced by a long-term culture significantly decreases adipogenic, osteogenic, and chondrogenic potential of the culture-aged TMSCs.

### Phenotypic characterization of TMSCs

**Fig. 4:**
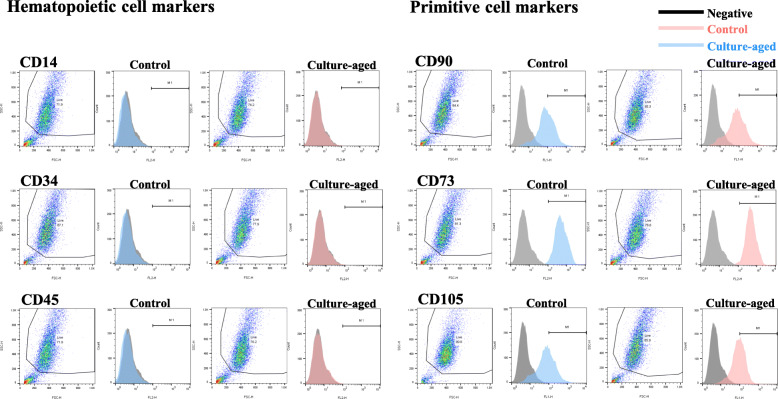
Changes in expression of stem cell surface markers in TMSCs with serial passaging. Surface markers of TMSCs were characterized using FACS analysis. Both hematopoietic (CD14, CD34, and CD45) and primitive (CD90, CD73, and CD105) cell surface markers were analyzed. Control- and culture-aged TMSCs were marked as red and blue lines, respectively

The phenotypic characteristics of replicative senescence from the culture-aged TMSCs were assessed by looking at the expression of representative stem cell surface markers, including hematopoietic cell markers (CD14, CD34, and CD45) and primitive cell markers (CD90, CD73, and CD105) (Fig. 4). By using FACS, we detected no significant changes in the expression of either hematopoietic or primitive surface protein markers between the control and culture-aged TMSCs (Fig. 4). Control (red line) and culture-aged TMSCs (blue line) results completely overlapped, indicating that senescent cells within culture-aged group cannot be distinguished by using these markers. The percentages of cells expressing each cell surface antigen are summarized in Table 1.

**Table 1:** Percent changes in the expression of stem cell surface markers in TMSCs with serial passaging. Both hematopoietic and primitive cell markers were determined using FACS analysis, and changes in surface markers were evaluated

Percentage	CD14	CD34	CD45	CD73	CD90	CD105
**Control**	8.45 ± 0.06	6.49 ± 0.10	8.63 ± 0.14	130.10 ± 7.51	555.09 ± 7.26	61.67 ± 1.70
**Culture-aged**	8.55 ± 0.16	6.55 ± 0.05	8.64 ± 0.06	118.90 ± 3.42	332.45 ± 14.21	54.96 ± 1.03
**Significance**	NS	NS	NS	NS	NS	NS

### Transcriptomic profiles of control and culture-aged TMSCs

**Fig. 5:**
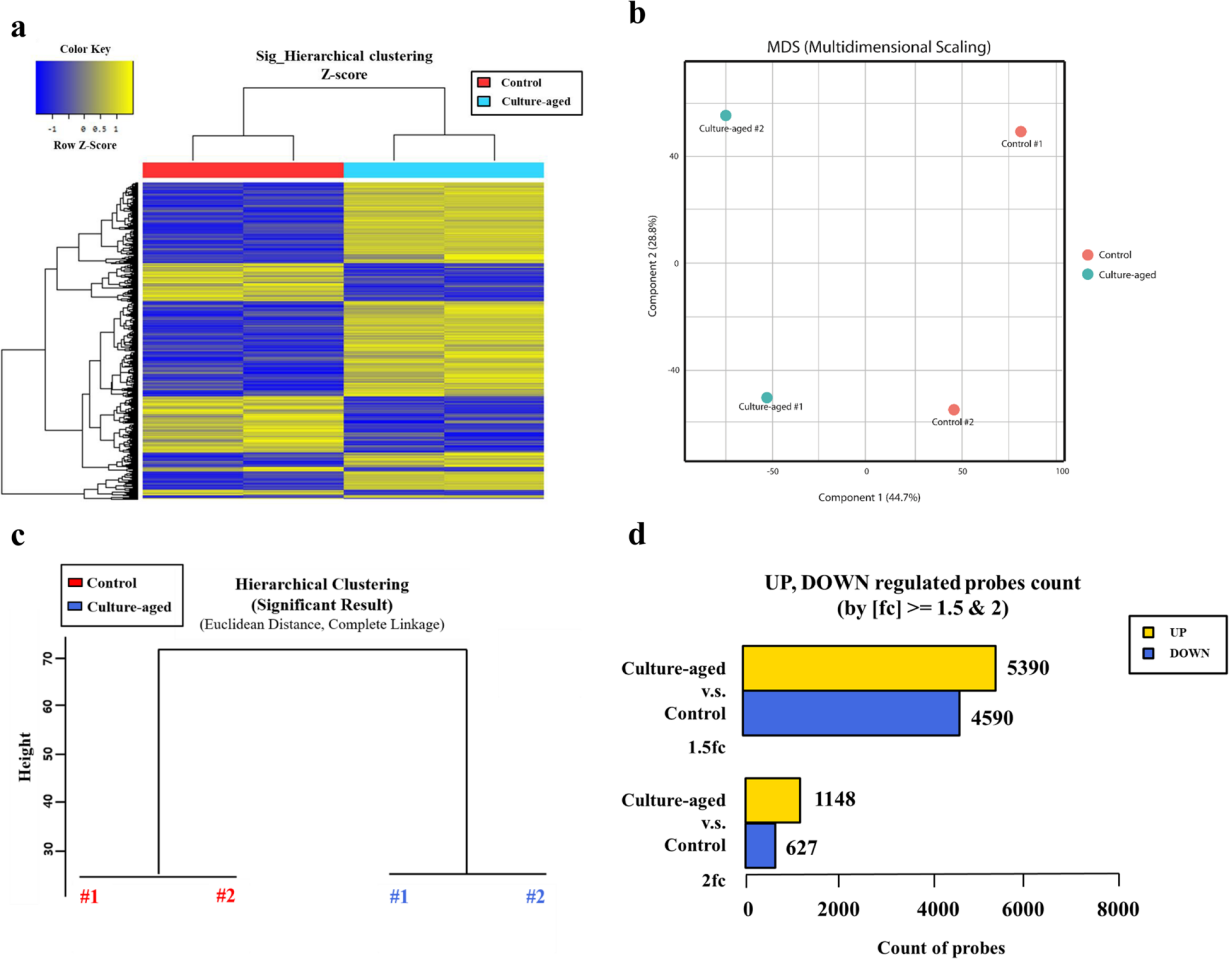
Transcriptomic profiles of control vs culture-aged TMSCs. An analysis of RNA whole-genome sequence data from control and culture-aged TMSC is presented. **a** A heatmap of hierarchical clustering analysis results indicate differentially expressing genes (rows) between the control and culture-aged TMSCs. Yellow and blue bars indicate upregulated and downregulated genes, respectively, in the culture-aged TMSCs in comparison to the control. For all comparisons, changes in gene expression are depicted as a heatmap. **b** MDS plots for microarray gene expression data compared control and culture-aged TMSCs. **c** Dendrogram depicting the results of hierarchical clustering analysis of the interclass correlation between control and culture-aged TMSCs, confirming classification of an interclass between the two groups. **d** The number of upregulated or downregulated genes between control and culture-aged TMSC groups with fold changes of at least 1.5 (1.5fc) or 2 (2.0fc) are presented

We next performed a transcriptomic analysis of the control and culture-aged TMSCs. A total of 18,164 genes showed significant differences between the two experimental groups were identified. Transcriptomic differences between control and culture-aged TMSCs are presented as a log_2_ fold-change heatmap distribution (|log_2_ (fold-change)| > 2; Fig. 5a). The quality of DNA-seq data was confirmed by a repeatability check using Pearson’s correlation coefficient analysis (*p* < 0.05). Based on multidimensional scaling (MDS) scores, the culture-aged group was distinguishable from the control (Fig. 5b).

Unsupervised hierarchical clustering showed a clear distinction between the control TMSCs and culture-aged TMSC (Fig. 5c). A subsequent evaluation of upregulated and downregulated genes in the two experimental groups (using a fold-change of 1.5 (1.5fc) as a cutoff) showed 5390 upregulated genes and 4590 downregulated genes in the culture-aged compared to the control TMSCs. When a 2-fold change (2fc) was applied as a cutoff, 1148 upregulated genes and 627 downregulated genes were identified (Fig. 5d).

### Functional enrichment analysis of highly regulated genes in culture-aged TMSCs

**Fig. 6:**
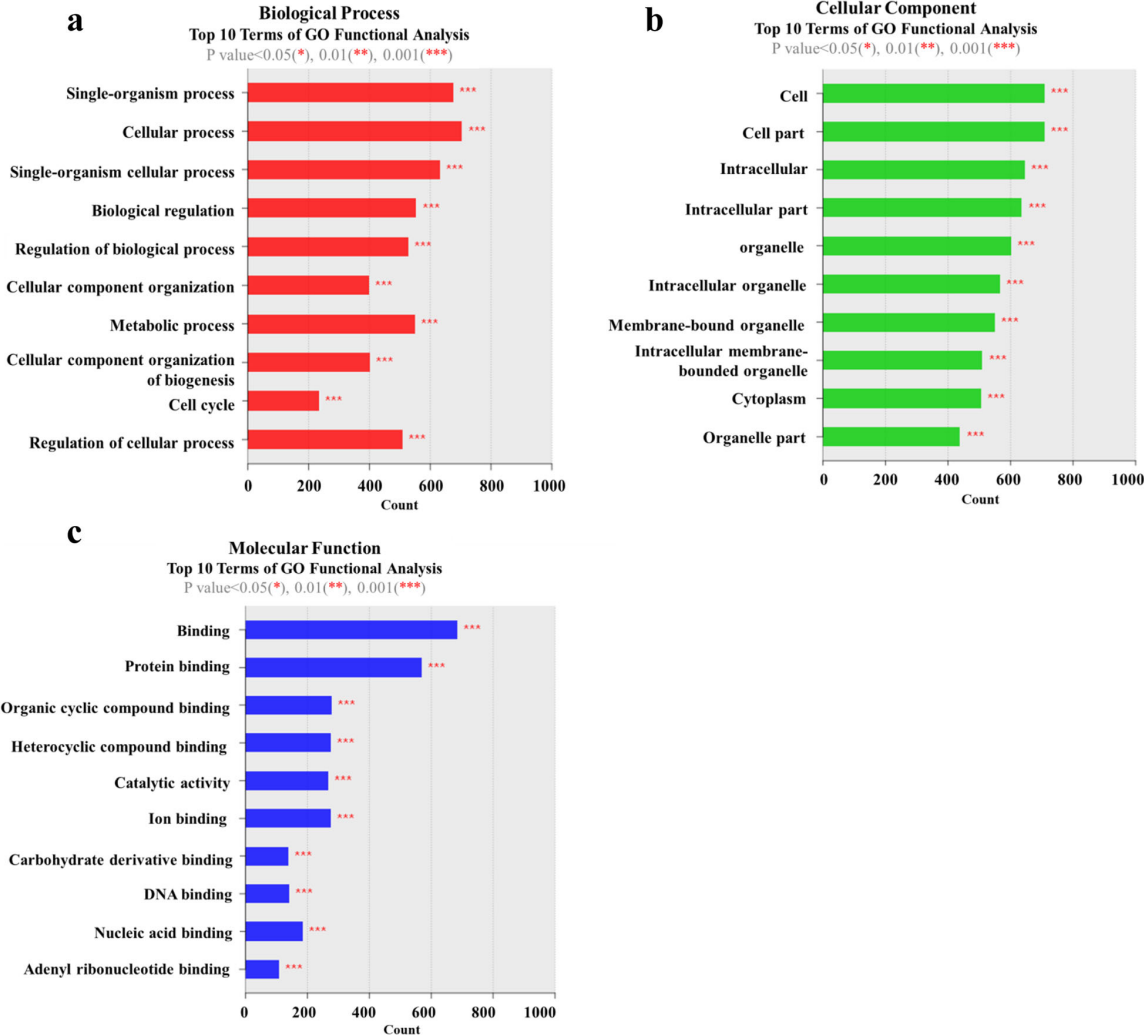
Functional classification of GO enrichment clusters. The distribution of GO terms for DEGs between control and culture-aged TMSCs were annotated according to the ontology categories, **a** biological process (BP), **b** cellular component (CC), and **c** molecular function (MF). *X*- and *Y*-axes indicate the number of DEGs and GO terms gene classification, respectively (***p < 0.001)

We next categorized highly differentially expressed genes from control and culture-aged TMSCs according to the following Gene Ontology (GO) classification terms: biological process (BP; Fig. 6a), cellular component (CC; Fig. 6b), and molecular function (MF; Fig. 6c). The top 10 enriched BP terms were single-organism process, cellular process, single-organism cellular process, biological regulation, regulation of biological process, cellular component organization, metabolic process, cellular component organization of biogenesis, cell cycle, and regulation of cellular process. The top 10 enriched CC terms were cell, cell part, intracellular, intracellular part, organelle, intracellular organelle, membrane-bound organelle, intracellular membrane-bounded organelle, cytoplasm, and organelle part. The top 10 enriched MF terms were binding, protein binding, organic cyclic compound binding, heterocyclic compound binding, catalytic activity, ion binding, carbohydrate derivative binding, DNA binding, and nucleic acid binding. A complete list of all genes of each GO term enrichment category is presented in Additional file 1.

### KEGG pathway analysis

**Fig. 7:**
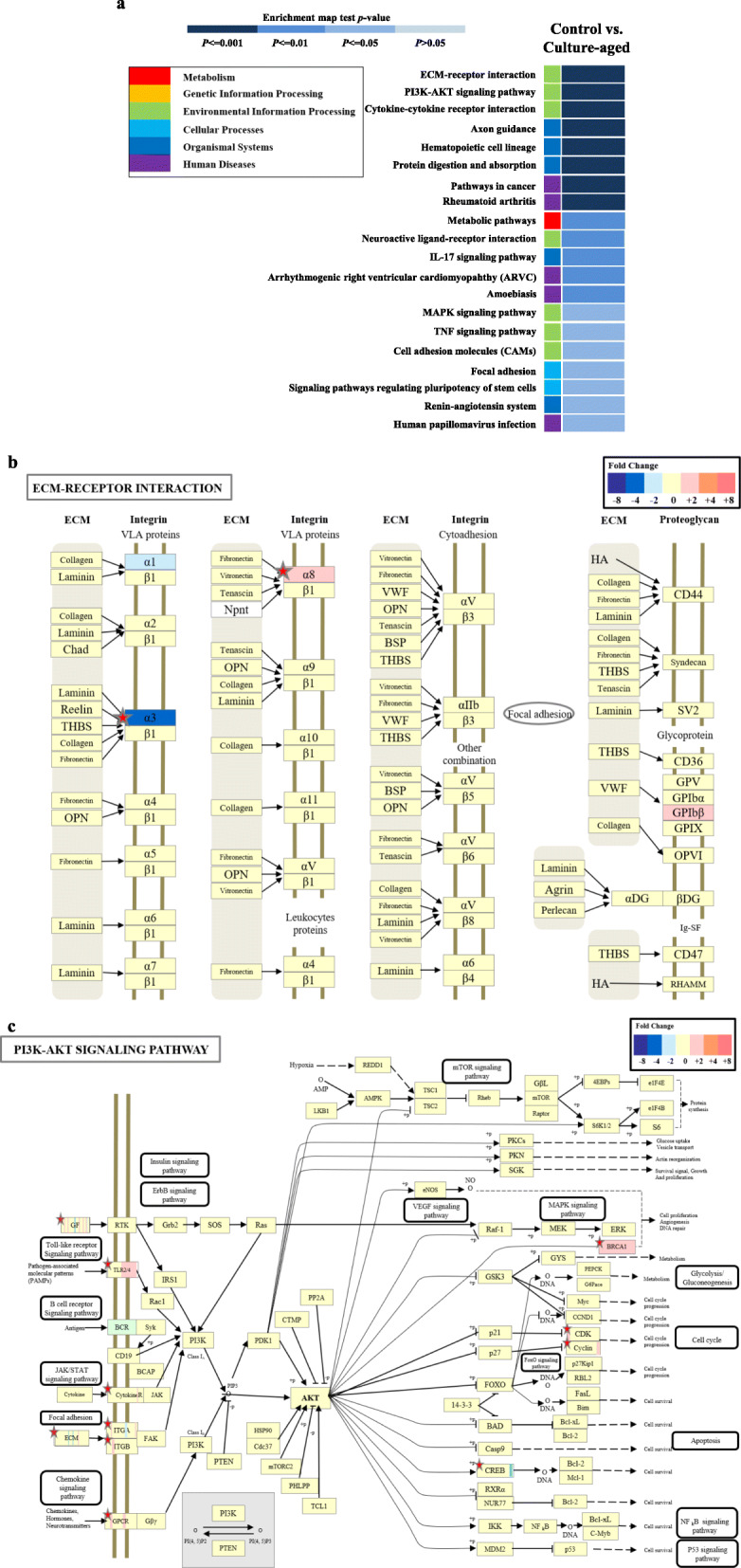
KEGG pathway classification map of the experimental group. Upregulated and downregulated genes were categorized into enriched functional signaling pathways. **a** All KEGG pathways were first classified into six categories: metabolism (red box), genetic information processing (yellow box), environmental information processing (green box), cellular processes (light blue box), organismal systems (blue box), and human diseases (purple box). These categories were then sub-categorized according to the level of significance. The significance of enrichment maps of these second categories is presented as blue color density. **b**, **c** Changes in expression of genes related to the ECM-receptor interaction pathway and PI3K-AKT signaling pathway with serial passaging were evaluated by KEGG enrichment map analysis

To evaluate signaling pathways associated with enriched genes in replicative senescence of culture-aged TMSCs, we performed a KEGG pathway analysis, which includes metabolism, genetic information processing, environmental information processing, cellular processes, organismal systems, and human disease (Fig. 7a). The following eight map categories were identified to be significantly different between the control and culture-aged TMSCs: ECM-receptor interaction, PI3K-AKT signaling pathway, cytokine-cytokine receptor interaction, axon guidance, hematopoietic cell lineage, protein digestion and absorption, pathway in cancer, and rheumatoid arthritis. Detailed gene profiles of the eight map categories are listed in Table 2.

**Table 2:** Genes included in the top eight sub-categories of the KEGG pathway map. All genes in the top eight sub-categories that showed the most significance (*p* < 0.001) of the KEGG pathway in Fig. 7a are listed. Genes that were upregulated (+) or downregulated (−) relative to the control group are listed in each sub-category

Map name	No of gene symbol	Gene symbol	Upregulated genes (control TMSC/culture-aged TMSC fc. (+))	Downregulated genes (control TMSC/culture-aged TMSC fc. (−))	***p*** value
Axon guidance	13	*PLXNA2, EPHA3, ROBO2, EPHB1, BMPR1B, EPHA5, EFNA5, SEMA6A, SLIT3, SEMA3E, SEMA3A, PAK3, TRPC4*	*PLXNA2, EPHA3, ROBO2, EPHB1, BMPR1B, EPHA5, EFNA5, SEMA6A, SEMA3E, SEMA3A, PAK3,*	*SLIT3, TRPC4*	4.88E-07
PI3K-Akt signaling pathway	23	*LPAR3, ITGA6, COL6A6, PDGFC, VEGFC, FGF5, IL7R, EFNA5, CREB5, CHRM2, HGF, RELN, CCNE2, TLR4, ITGA8, PDGFD, CDK2, ITGA7, KITLG, BRCA1, ITGB3, LAMA3, LAMA1*	*LPAR3, ITGA6, PDGFC, VEGFC, FGF5, IL7R, EFNA5, CHRM2, RELN, CCNE2, TLR4, ITGA8, CDK2, KITLG, BRCA1, ITGB3, LAMA1*	*COL6A6, CREB5, HGF, PDGFD, ITGA7, LAMA3*	7.17E-12
Pathways in cancer	32	*CKS1B, TGFB2, SLC2A1, PTGER3, LPAR3, GSTM5, ITGA6, CASP8, AGTR1, HHIP, MAPK10, LEF1, VEGFC, FGF5, IL7R, HGF, CCNE2, DAPK1, CKS2, MAPK8, ETS1, CDK2, KITLG, BRCA2, BDKRB2, RAD51, BIRC5, LAMA3, LAMA1, NOTCH3, E2F1, PLCB1*	*CKS1B, TGFB2, SLC2A1, LPAR3, GSTM5, ITGA6, AGTR1, HHIP, LEF1, VEGFC, FGF5, IL7R, CCNE2, CKS2, MAPK8, ETS1, CDK2, KITLG, BRCA2, RAD51, BIRC5, LAMA1, E2F1*	*NOTCH3, PLCB1, PTGER3, CASP8, MAPK10, HGF, DAPK1, BDKRB2, LAMA3*	2.02E-14
ECM-receptor interaction	11	*ITGA6, SDC1, COL6A6, HMMR, RELN, ITGA8, ITGA7, ITGB3, LAMA3, LAMA1, GP1BB*	*ITGA6, SDC1, HMMR, RELN, ITGA8, ITGB3, LAMA1, GP1BB*	*COL6A6, ITGA7, LAMA3*	3.22E-08
Protein digestion and absorption	6	*COL3A1, DPP4, COL6A6, ELN, COL14A1, SLC1A1*	*ELN*	*COL3A1, DPP4, COL6A6, COL14A1, SLC1A1*	0.002749
Rheumatoid arthritis	5	*TGFB2, ATP6V1E2, TLR4, MMP3, TNFSF13B*	*TGFB2, ATP6V1E2, TLR4, MMP3*	*TNFSF13B*	0.015019
Hematopoietic cell lineage	6	*IL1R1, ITGA6, IL7R, KITLG, ITGB3, GP1BB*	*ITGA6, IL7R, KITLG, ITGB3, GP1BB*	*IL1R1*	0.003733
Cytokine-cytokine receptor interaction	15	*TGFB2, TNFSF4, IL1R1, ACKR3, IL1RAP, TNFSF10, BMPR1B, PDGFC, VEGFC, IL7R, CCL26, HGF, TNFRSF11B, KITLG, TNFSF13B*	*TGFB2, TNFSF4, IL1RAP, BMPR1B, PDGFC, VEGFC, IL7R, TNFRSF11B, KITLG*	*IL1R1, ACKR3, TNFSF10, CCL26, HGF, TNFSF13B*	1.34E-06

To place these results in the context of morphological changes in culture-aged TMSCs and their proliferation rate, we focused on changes in cellular membrane-related gene profiles, namely ECM-receptor interaction and the PI3K-AKT signaling pathway. A total of 11 ECM-receptor interaction-related genes (*ITGA6*, *SDC1*, *COL6A6*, *HMMR*, *RELN*, *ITGA8*, *ITGA7*, *ITGB3*, *LAMA3, LAMA1, GP1BB*) and 23 PI3K-AKT signaling pathway-related genes (*LPAR3*, *ITGA6*, *COL6A6*, *PDGFC*, *VEGFC*, *FGF5*, *IL7R*, *EFNA5*, *CREB5*, *CHRM2*, *HGF*, *RELN*, *CCNE2*, *TLR4*, *ITGA8*, *PDGFD*, *CDK2*, *ITGA7*, *KITLG*, *BRCA1*, *ITGB3*, *LAMA3*, *LAMA1*) were identified (Table 2).

### Expression of the ECM-receptor protein, integrin, and related signaling

The identified genes related to ECM-receptor interaction from the in silico analyses were integrin α1, α3, α8, and β3, all of which are a member of the integrin family. A plot of ECM-receptor-related KEGG pathway showed that integrin α1 (− 2fc) and α3 (− 4fc) were significantly lower in the culture-aged TMSCs compared to the control (Fig. 7b). In contrast, integrin α8 was significantly higher in the culture-aged TMSCs (+ 2fc). All changes in the expression of integrin α and β isotypes are listed in Table 3 and Table 4, respectively. In contrast to ECM-receptor-related genes (Fig. 7b), a plot of PI3K-AKT signaling pathway from in silico analyses showed no significant changes in the expression of PI3K and AKT (Fig. 7c).

**Table 3:** Integrin *α* subunit genes analyzed by transcriptomic analysis. All 17 integrin *α* subunit genes are listed based on transcriptomic analysis. Fold changes (fc) and local-pooled-error (LPE) test scores of culture-aged groups in comparison to control groups are listed

Description	Control TMSC/culture-aged TMSC fc.	Control TMSC/culture-aged TMSC.LPE.stat	***p value***
Integrin, alpha 2 (CD49B)	1.429960	2.918061868	0.122394203
Integrin alpha 2b	− 1.057931	− 0.291252427	1
Integrin alpha 3	− 1.505534	− 3.179636945	0.06438614
Integrin alpha 4	1.321261	1.765683601	0.733428278
Integrin alpha 5	− 1.372114	− 3.000065996	0.101444605
Integrin alpha 6	4.054390	9.559895029	0
Integrin alpha 7	− 2.289401	− 5.295561818	1.84079E-05
Integrin alpha 8	2.124782	6.653867623	7.72016E-09
Integrin alpha 9	1.150962	0.644712505	1
Integrin alpha 10	− 1.009541	− 0.052283863	1
Integrin alpha 11	− 1.345036	− 1.453086794	0.8953197
Integrin alpha V	− 1.459784	− 3.155850719	0.068660472
Integrin alpha E	1.200017	1.240547522	0.977341438
Integrin alpha L	1.004449	0.021283939	1
Integrin, alpha M	1.232395	1.015699765	1
Integrin alpha X	− 1.258408	− 1.407297271	0.915210992
Integrin alpha FG-GAP repeat containing 2	1.330372	1.993520189	0.587179204

**Table 4:** Integrin *β* subunit genes analyzed by transcriptomic analysis. All 12 integrin *β* subunit genes are listed based on transcriptomics analysis. Fold changes (fc) and local-pooled-error (LPE) test scores of culture-aged groups compared with control groups are listed

Description	Control TMSC/culture-aged TMSC fc.	Control TMSC/culture-aged TMSC. LPE. stat	*P-value*
Integrin beta 1	-1.018912	-0.295268866	1
Integrin beta 1 binding protein 1	1.805971	3.486672385	0.026933575
Integrin beta 1 binding protein 2	-1.043040	-0.184731293	1
Integrin beta 3	2.019125	4.370971051	0.001212392
Integrin beta 3 binding protein	4.309648	8.27039277	1.05026E-13
Integrin beta 4	1.066900	0.28484834	1
Integrin beta 5	1.071176	0.535597735	1
Integrin beta 6	1.377460	1.383266915	0.924165462
Integrin beta 6	-1.179610	-0.704547478	1
Integrin beta 7	1.198288	0.915917305	1
Integrin beta 8	1.722740	3.711656951	0.013329454
Integrin beta like 1	-1.892502	-5.617097637	3.41471E-06

**Fig. 8:**
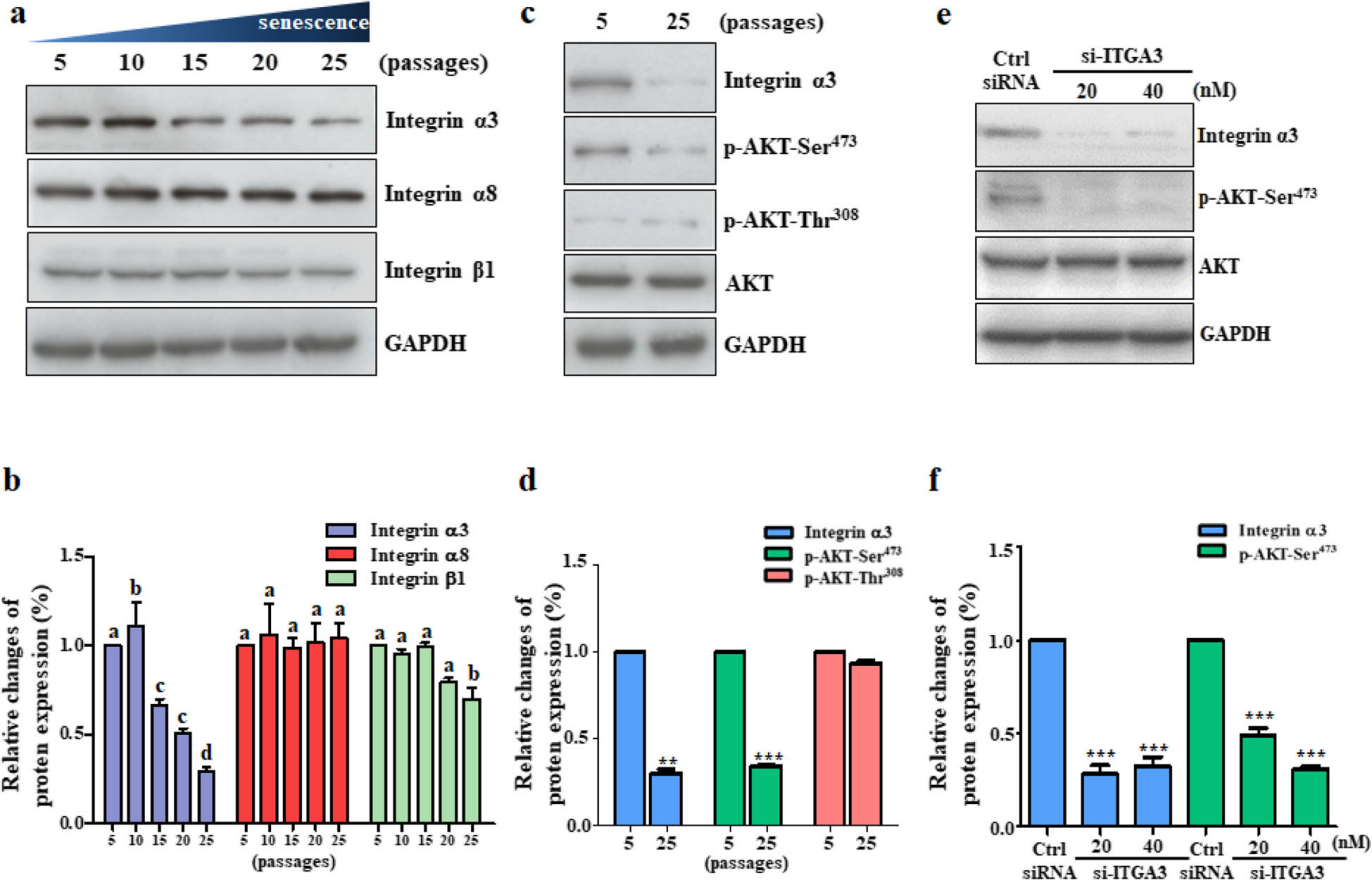
Decreased ITGA3 and AKT protein expression in culture-aged TMSCs. **a**, **b** Changes in the expression of integrin family proteins induced by serial passaging were confirmed by Western blot analyses. **c**, **d** Changes in total and phosphorylated AKT protein expression between control (5 passage) and culture-aged TMSCs (25 passage) were also assessed. **e** Control TMSCs were transfected with either ITGA3 siRNA or control siRNA. **b**, **d**, **f** Bar graph represents relative changes in protein expression, determined by the intensity of each band normalized to that of the respective GAPDH band. The *p* values were considered statistically significant at the *p* < 0.05 (*), *p* < 0.01 (**), and *p* < 0.001 (***), and significant differences among experimental groups were indicated with different alphabetical letters infigures

The genes identified from in silico KEGG pathway analysis were validated by performing Western blot analyses on integrin α/β isotypes and molecules involved in the PI3K-AKT signaling pathway (Fig. 7c); these include ITGA3, ITGA8, and ITGB1, total AKT, Ser473-phosphorylated AKT (p-AKT-Ser^473^), and Thr308-phosphorylated AKT (p-AKT-Thr^308^) (Fig. 8a–d).

As replicative senescence of the TMSCs progresses with serial passaging, the protein expression of ITGA3 significantly decreased, especially after passage 15 (Fig. 8a, c), whereas there were no significant changes in the protein expression of ITGA8 and ITGB1 (Fig. 8a, c). In addition, total AKT protein expression was not altered with the serial-passaging of TMSCs but p-AKT-Ser^473^ was significantly decreased with the serial-passaging (Fig. 8c).

To validate potential relationship between ITGA3 and p-AKT-Ser^473^, we knockeddown the ITGA3 by transfecting TMSCs with ITGA3 siRNA. As expected, transfection with ITGA3 siRNA significantly decreased protein expression of p-AKT-Ser^473^ (Fig. 8e, f; *p* < 0.001), suggesting that ITGA3 regulates the phosphorylation of AKT on Ser^473^.

Collectively, these results suggest that long-term passaging of TMSCs decreases ITGA3 signaling, which reduces the phosphorylation of AKT on Ser^473^, consequently decreasing AKT activity and cell proliferation of TMSCs (Fig. 8a–f).

## Discussion

Therapeutic applications of MSCs have increasingly been popular in regenerative medicine. MSCs have shown increasing promise for therapeutic applications, yet information on specific MSC-molecular markers and effects of culture expansion on the functional characteristics of MSCs has remained limited [36]. Previously, we isolated MSCs from tonsillar tissue of children who had undergone a tonsillectomy [37]. TMSCs have been implied for various tissue engineering of the bone [18, 38], parathyroid gland [17], pancreatic islet [39], Schwan cells [22], tendon [21], nerve cell [20, 23], and skeletal muscle [19] regeneration. For practical applications of cell therapy, it is necessary to obtain a sufficient number of cells through a long-term serial culture [38]. A transcriptomic study conducted by our colleagues revealed that TMSCs are highly enriched for genes that could result in high proliferative and interactive [40]. The stemness characteristics of TMSCs including their doubling time, and surface markers could be well maintained with serial passages [13]. However, there is a possible risk of stem cell heterogeneity, where senescent cells within a TMSC pool might have lost their stemness, decreasing therapeutic efficacy. Complicating quality control of stem cells for therapeutic use. To resolve these issues, this study sought to investigate changes in transcriptomic characteristics of the senescent human TMSCs induced by serial passaging and to identify a novel senescence-specific biomarker.

In our study, we found that the culture-aged senescent TMSCs exhibit senescence-specific characteristics, including delayed cell proliferation (Fig. 2a), an enlarged cell morphology (Fig. 2b–d), increased senescent-stained cells (Fig. 2d, e), and decreased telomere length regulating factor gene 1 (Fig. 2f), stemness, and self-renewability (Fig. 3a–d). This phenomenon could probably be described as “Hayflick phenomenon” [41], where the cells, specifically TMSCs in this study, lose their proliferation competency and original cell morphology [24, 42]. In fact, the expression of telomere-related gene known as TRF-1 as well as the doubling time of TMSCs was decreased with the increased passage number. Nevertheless, since the telomere length of the TMSCs was not directly assessed in this study, this is only speculation at this point, and therefore, a further study on telomere length would be necessary to validate the Hayflick phenomenon observed in this study.

The replicative senescence induced by the serial culture of TMSCs decreased not only replicative capacity but also the differentiation potential of TMSCs. An examination of three mesodermal differentiation potentials—adipogenesis, osteogenesis, and chondrogenesis—revealed that early-passage TMSCs (passages 5–8) have a superior differentiation potential compared with late-passage TMSCs (passages 20–25). It has also been reported that the quality of other MSCs, such as adipose tissue- [43], bone marrow- [36], and Wharton’s jelly-derived MSCs [2], were also shown to progressively decline with donor age [27]. The serial long-term culture of TMSCs may not necessarily have the equal effects as the donor age, yet the serial culture could potentially decrease proliferation capacity as well as telomere length, simulating the effects of replicative senescence. These observations underscore the need to evaluate and identify genetic profiles, especially as they relate to serially passaged TMSCs.

Our initial observation found that the hematopoietic and primitive markers between the control and culture-aged TMSCs remained to be unchanged. Our inability to detect changes in the expression of stem cell surface markers with senescence (Fig. 4) implied that more precise criteria are needed for identifying senescent serial-passaged cells. A transcriptomic approach has been provided greater discriminating power in establishing biomarkers for various applications, and therefore, we conducted genomic/transcriptomic analyses to uncover a new biomarker.

To identify a specific biomarker, we first analyzed changes in whole-transcriptome expression profiles. The transcriptomic analyses showed that genes involved with regulation of cellular process, membrane-bound organelle, intracellular membrane-bounded organelle, and protein binding-related genes were the most significantly altered with the serially passaged TMSCs. Of the transcripts identified from transcriptomic analyses, those involved in ECM-receptor interaction, specifically integrin molecules, were chosen as potential biomarker candidates of replicative senescence (Fig. 7a), because the integrin family has been known as a representative cell membrane protein that can regulate cell-cell adhesion and interaction [29]. There are 24 known integrin heterodimers comprised of one of 18 *α* subunits and one of 8 *β* subunits; these integrin heterodimers mediate a diverse range of functions, including cell-cell adhesion [29]; growth factor receptor responses; and intracellular signaling cascades for cell migration [44], differentiation [45, 46], survival [47], and proliferation [32]. The major extracellular ligands for each heterodimer include collagen (α1, α2, α10, and α11), leukocyte-specific ligands (α4, α9, αE, αM, αL, αD, αX, β2, and β7), RGD (α5, α8, αIIIb, αv, β3, β5, β6, and β8), and laminin (α3, α6, α7, and β4) [32]. Among many integrin molecules related to replicative senescence, integrin α3 (ITGA3) and α8 (ITGA8) showed the most significant changes with the culture-aged TMSCs. Western blot analyses also confirmed that the protein expression of ITGA3 progressively declined with the passage number, indicating its potential association with the stemness of TMSCs.

Our study identified integrin α3 (*ITGA3*) as a significantly decreased cell membrane-bound protein, which affects phospho-AKT-mediated signaling to alter cellular process in senescent TMSCs. These findings suggest that a transcriptomic approach could provide greater discriminating power in establishing new senescence biomarkers for studying MSC. During development, many cellular responses are initiated by integrin signaling induced by the local ECM [48]. Among genes that were significantly differentially expressed, α3/β1-integrin is annotated as being involved in ECM-receptor interaction [49], regulation of actin [50], pathway in cancer [51, 52], focal adhesion [53], and Alzheimer’s disease [54]. The findings from our study propose that α3 β1-integrin could also be used as an indicator for replicative senescence of stem cells, particularly for TMSCs, because the expression of α3β1-integrin decreases as the age of TMSCs progresses. In fact, Tomellini et al. (2019) reported that the ITGA3^+^ cell population exhibited greater HSC-specific features than the previously established Lin-CD34^+^CD38^−^CD45RA^−^CD90^+^CD49f^+^ phenotype, especially when the HSCs were cultured for a long term. The authors also pointed out ITGA3 as a sufficient biomarker to separate primitive EPCR^+^CD90^+^CD133^+^CD34^+^CD45RA^−^ HSC population into two functionally distinct fractions depending on their culture duration. In particular, the ITGA3^+^ cells exhibited greater multilineage differentiation potential, serial reconstitution ability, and long-term engraftment of the cord blood [55]. Our results validate this previous study, where ITGA3 could be used as a potential biomarker of stem cells retaining full stemness and multipotency characteristics.

It is also interesting to note that a similar pattern was observed with its neighboring beta-subunit of integrin, integrin β1 (ITGB1), progressively decline with serial passages, even though the reduction is less dramatic compared to ITGA3. ITGA3 regulates several cell activities, including adhesion and proliferation, whereas the ITGB1 regulates cell behaviors [56]. Considering that replicative senescence induced by serial passages is likely to put more pressure on the proliferation of TMSCs, it is probably expected that ITGA3 is more prone to changes upon the stresses related to replicative senescence. In addition to the decreased expression of both ITGA3 and ITGB1, we also found lower expression of AKT as well as phosphorylation of threonine-308 and serine-473 from the TMSCs at 25th passages compared to the early passage TMSCs. AKT pathway is known as a downstream transcript of integrin signaling pathways, and our study also validated the involvement of ITGA3 expression on the AKT signaling pathway, where knockdown of ITGA3 decreased the phosphorylation of AKT at serine-473. Considering that AKT is an important regulator of cellular activity, the replicative senescence of TMSCs simulated by a long-term serial passage in this study seems to reduce the expression of whole α3β1-integrin, consequently decreasing the PI3K-AKT activity as well as the stemness of the TMSCs.

Along with ITGA3 as being a negative marker of senescence, we screened other ECMs that could be used as a positive marker of senescent TMSCs. Other isotypes of integrins *α* and *β* were not significantly altered with the long-term culture of TMSCs. We also sought to find a more reliable specific biomarker for senescent TMSCs for use in isolating these cells and distinguishing them from other cells in a heterogeneous population. The in silico analyses showed that the expression of most of α isotypes (α2, α4, α5, α6, α7, α9, α10, α11, and αV) was unchanged except for ITGA3 and ITGA8 (Fig. 7b). When we checked protein expression changes of ITGA3 and ITGA8, we found that significantly decreased ITGA3 expression and unchanged ITGA8 expression were detected with the serial passages.

To gain insight into the role of ECM-receptor interactions in replicative senescence, we analyzed changes in expression profiles of TMSCs and identified α3β1-integrin, especially ITGA3, as a potential biomarker of senescent TMSCs. α3β1-integrin could be used as a reliable biomarker that can be applied in the senolytic technique for removing senescent cells from a heterogeneous pool of TMSCs, thereby improving the efficacy of TMSCs for regenerative therapy.

To date, the identification of unique markers that unequivocally detect and quantify senescent cells remains challenging and various studies are underway to address them, such as senolytic techniques [35]. While the repurposed senolytics usually have on-target and/or off-target toxicities. Therefore, strategies to reduce the toxicity of senolytics or another sensitizing culture-aged cell screening technique are urgently needed. To achieving a high number of TMSCs for clinical use, further technologies such as larger culturing or bioreactor techniques after excluding culture-aged cells will also be needed. Additionally, the new biotechnology to achieve high MSC numbers such as using bioreactors and overexpression of human telomerase transcriptase (hTERT) should be chosen for further clinical applications [41].

## Conclusion

In the current study, we investigated transcriptomic changes in long-term-cultured TMSCs compared with the short-term cultured cells. We identified a novel cellular protein ITGA3, which contributes to the decreased AKT activity and consequently decreasing cell proliferation of TMSCs. ITGA3 could be used as an effective indicator or marker to improve quality control during isolation and expansion of MSCs, thereby increasing the therapeutic efficacy of MSC-based regenerative therapy. ITGA3 could be used for selecting other MSCs types that hold high stemness multi-differentiation potential. Future studies should investigate detail molecular mechanism including changes of intracellular localizations of ITGA3 to other MSCs control senescence. In addition to the *ITGA3*, other molecules classified into different GO term categories and related regulatory mechanisms remain to be identified.

## Supplementary information

**Additional file 1.** A complete list of all genes that could be included in each GO term enrichment category is presented. The top 10 enriched biological processes, cellular component, and molecular functions related terms were listed.

## Data Availability

The datasets used and/or analyzed during the current study are available from the corresponding author on reasonable request. Ethics approval and consent to participate This research has been approved by the Ethics Committee of Ewha Womans University Mokdong Hospital.

## Abbreviation

A/A: Antibiotics/antimycotics
ANOVA: Analysis of variance
BP: Biological process
CC: Cellular component
DEGs: Differentially expressed genes
ECM: Extracellular matrix
ECL: Enhanced chemiluminescence
ESCs: Embryonic stem cells
FBS: Fetal bovine serum
FC: Functional component
FITC: Fluorescein isothiocyanate
ITGA3: Integrin α3
iPSCs: Induced pluripotent stem cells
KEGG: Kyoto encyclopedia of genes and genomes
PBS: Phosphate-buffered saline
PE: Phycoerythrin
P/S: Penicillin/streptomycin
RMA: Robust multi-average
SA-β-gal: Senescence-associated β-galactosidase
SDS-PAGE: Sodium dodecyl sulfate polyacrylamide gel electrophoresis
TMSCs: Tonsil-derived mesenchymal stem cells
